# Tissue Doppler Imaging in Acute and Critical Care: Enhancing Diagnostic Precision

**DOI:** 10.3390/medicina61061051

**Published:** 2025-06-06

**Authors:** Ugo Giulio Sisto, Daniele Orso, Davide Maione, Francesco Venturelli, Antonio De Luca

**Affiliations:** 1 Department of Emergency Medicine, Azienda Sanitaria Universitaria Giuliano Isontina (ASUGI), University Hospital of Trieste, 34149 Trieste, Italy; 2 Department of Emergency, Azienda Sanitaria Universitaria Friuli Centrale (ASUFC), University Hospital “Santa Maria della Misericordia”, 06156 Udine, Italy; 3 Cardiothoracovascular Department, Azienda Sanitaria Universitaria Giuliano Isontina (ASUGI), University Hospital of Trieste, 34149 Trieste, Italy

**Keywords:** tissue Doppler imaging, acute care, diastolic dysfunction, point-of-care ultrasound, echocardiography, emergency medicine, intensive care

## Abstract

*Background and Objectives:* The introduction of portable ultrasound devices has transformed clinical practice in emergency medicine. Diagnostic accuracy and patient safety have been enhanced by point-of-care ultrasonography (POCUS), which has become a fundamental diagnostic and procedural tool. In addition to the standard clinical evaluation, POCUS provides quick patient assessments, allowing for the exclusion of life-threatening conditions and prognostication in different critical situations. Tissue Doppler imaging (TDI), as an advanced echocardiographic technique, offers additional quantitative data by measuring myocardial velocities, thereby improving the evaluation of systolic and diastolic ventricular function. The purpose of this review is to highlight the potential use of TDI in multiple acute and critical conditions. *Materials and Methods:* We conducted a narrative review of the main application topics for TDI. *Results:* TDI is an essential diagnostic and prognostic tool for acute coronary syndromes, assessing systolic or diastolic dysfunction, and etiological diagnosis of acute heart failure. It helps differentiate cardiogenic pulmonary edema from acute respiratory distress syndrome and identifies right ventricular systolic dysfunction in acute pulmonary embolism. TDI also facilitates distinctions between hypertension emergencies and urgencies and contributes to the stratification of atrial fibrillation reoccurrence risk. Furthermore, it aids in the differentiation of constrictive pericarditis from other restrictive cardiomyopathy patterns. In intensive care settings, TDI is particularly valuable during mechanical ventilation weaning, where elevated E/E’ values serve as a predictor of weaning failure. Due to its accessibility, rapid execution, and high reproducibility, it is suitable for longitudinal monitoring. *Conclusions:* TDI enhances the diagnostic precision, guides therapeutic strategies, and provides critical prognostic insights across a wide range of time-sensitive clinical scenarios, solidifying its role as an indispensable tool in modern emergency and critical care practice.

## 1. Introduction

The development of portable ultrasound devices over the past four decades has enabled clinicians to use ultrasounds across a wide range of clinical scenarios. Point-of-care ultrasound (POCUS) has evolved as an extension of the physical examination, enhancing diagnostic accuracy and becoming an essential tool for safely performing invasive procedures. In emergency medicine, POCUS helps to identify high-risk conditions, allowing for rapid and appropriate treatment, while also offering valuable prognostic information [1].

In this context, echocardiography plays a central role in the assessment of a wide range of clinical presentations (e.g., chest pain, dyspnea, and syncope), quickly confirming or ruling-out life-threatening conditions at the bedside, such as acute coronary syndrome, pulmonary embolism, heart failure, pericardial effusion with or without cardiac tamponade, and undifferentiated shock [2,3,4,5,6]. In addition to the standard evaluation, tissue Doppler imaging (TDI) may provide additional insights in several clinical scenarios, becoming a useful tool also in the field of emergency medicine. The aim of this review is to provide a summary of the current evidence regarding the use of TDI in critically ill patients, from the emergency department to the intensive care unit.

## 2. Materials and Methods

A narrative review was conducted to provide a comprehensive overview of the applications of tissue Doppler imaging (TDI) in acute and critical care settings. The main objective was to synthesize the existing literature concerning the diagnostic and prognostic value of TDI across a wide range of urgent clinical scenarios, including acute coronary syndromes, acute heart failure, pulmonary embolism, hypertensive crises, septic cardiomyopathy, atrial fibrillation, constrictive pericarditis, and weaning from mechanical ventilation.

### 2.1. Literature Search and Study Selection

An extensive search of the literature was carried out using electronic databases like PubMed, Embase, and the Cochrane Library, including all publications published between the study’s inception and May 2025. The search strategy combined key terms and Medical Subject Headings (MeSH) including “Tissue Doppler Imaging” (TDI), “acute care”, “critical care”, “emergency medicine”, “intensive care”, along with more specific disease-related keywords such as “acute coronary syndrome”, “acute heart failure”, “pulmonary embolism”, “hypertensive crisis”, “septic cardiomyopathy”, “atrial fibrillation”, and “constrictive pericarditis”. The search was enhanced by using Boolean operators and relevant synonyms.

Studies with relevant data on the use of TDI in the clinical settings of interest were identified after screening their titles and abstracts. The full texts of potentially relevant articles were subsequently retrieved and evaluated. In addition, reference lists of selected articles were manually reviewed to identify any further relevant studies. Given the narrative nature of this review, no formal quality assessment or meta-analysis was performed. Instead, the emphasis was placed on capturing a broad perspective of the emerging evidence.

### 2.2. Data Extraction and Synthesis

Multiple authors performed data extraction independently to gather critical study characteristics. The TDI parameters, diagnostic thresholds, prognostic implications, study design, and sample characteristics were the information collected. The findings from the selected studies were then synthesized qualitatively to provide an integrated discussion of the technical features and reproducibility of TDI, its clinical applications for rapid bedside diagnosis, its advantages and limitations in various acute settings, and the role of TDI parameters as predictors of adverse outcomes. By integrating heterogeneous findings, this narrative synthesis was able to create a cohesive assessment of TDI’s utility in improving the diagnostic precision and guiding therapeutic decisions in time-sensitive environments.

### 2.3. Ethical Considerations

As this review was based exclusively on previously published studies and publicly available data, no original human or animal research was performed, and therefore, ethical approval was not required.

## 3. Tissue Doppler Imaging Characteristics

TDI evaluates myocardial velocities during both contraction and relaxation of the ventricles via the Doppler effect of the reflected ultrasounds. Similar to the pulsed-wave Doppler technique, TDI measures systolic and diastolic velocities throughout the cardiac cycle, typically at the level of the mitral and tricuspid annuli in the apical four-chamber view. TDI can be performed quickly, easily, and reproducibly and provide quantitative information with established prognostic value [7]. TDI cannot differentiate whether the analyzed segment is in active contraction or passive movement due to the angle dependence (Table 1) [8].

**Figure 1:**
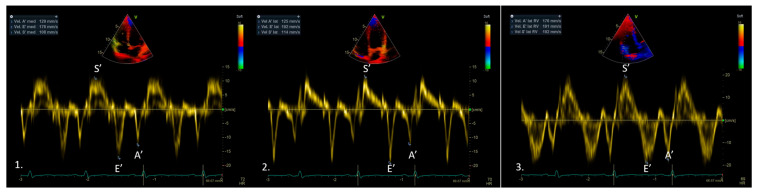
Tissue Doppler imaging (TDI) of a normal subject, obtained at the level of the inferior septum (1) and lateral wall (2) of the left ventricle, and the lateral wall of the right ventricle (3), showing normal systolic (S’) and diastolic (E’ and A’) velocities.

Current applications include the assessment of both biventricular systolic and diastolic function (Figure 1).

Left ventricular (LV) systolic function is assessed by measuring peak systolic mitral annular velocity (S’), which is associated with an ejection fraction (EF) greater than 50% when >5.4 cm/s, with an 88% sensitivity and 97% specificity [9]. Similarly, the peak systolic velocity of the tricuspid annulus can be used to evaluate the right ventricular (RV) systolic function, with a cutoff value of 9.5 cm/s [10]. TDI can be used for non-invasive estimation of LV filling pressure when assessing LV diastolic function. According to the guidelines, a septal mitral annular protodiastolic velocity (E’) < 7 cm/s or a lateral E’ < 10 cm/s along with a ratio of early diastolic transmitral flow velocity (E) to average E’ (E/E’) > 14 is indicative of diastolic dysfunction and elevated LV filling pressure [11].

## 4. TDI in Acute Coronary Syndrome (ACS)

Coronary artery disease (CAD) remains a major health concern around the world and is responsible for both morbidity and mortality [12,13]. CAD can lead to different degrees of cardiac dysfunction, including a reduction in both regional LV and global systolic function, as well as diastolic dysfunction, which are associated with major adverse cardiovascular events (MACEs) [14,15]. The diagnosis and prognostic stratification of these patients, both acute and chronic, require a precise assessment of LV function.

Acute coronary syndromes (ACS) encompass a spectrum of conditions characterized by symptoms and signs of acute cardiac ischemia [16]. In this context, transthoracic echocardiography is valuable for detecting signs of ongoing ischemia, previous myocardial infarction, and its associated complications [16]. In addition, several echocardiographic parameters, such as the reduction of LV ejection fraction (LVEF; <40%) and tricuspid annulus plane systolic excursion (TAPSE; <17 mm), the mitral inflow E-wave deceleration time, the E/E′ ratio, and the mitral regurgitation (MR) have been associated with increased MACEs and mortality in patients with ACS [16,17].

TDI has the potential to be a helpful tool for these patients. Systolic and early diastolic velocities (i.e., S’ and E’) can be used as early indicators of cardiac ischemia, often preceding the impairment of conventional echocardiographic parameters. Derumeaux et al. demonstrated that S’ and E’ are highly sensitive to ischemia, which typically causes a reduction in both velocities [18]. In contrast, the late diastolic velocity (i.e., A’) increases during acute ischemia [18]. This phenomenon likely results from the high energy demands of both systolic contraction and early diastolic relaxation, making them highly sensitive to interruptions in oxygen supply [19].

Beyond identifying myocardial ischemia, the impairment of systolic and diastolic TDI velocities can provide relevant prognostic information. In a cohort of patients with ST-elevation myocardial infarction, Biering-Sørensen et al. demonstrated a more than 2-fold increased risk of death, heart failure (HF), or new myocardial infarction in patients with reduced S’ and E’ velocities, independent of other conventional echocardiographic parameters [20].

The primary difference is that TDI determines the actual velocity of the myocardial tissue, while classic echocardiography primarily relies on visual assessments of wall motion. Objective, quantitative data from TDI in ACS can detect subtle regional dysfunction, changes in myocardial motion, and deformation which may not be visible in conventional echo [21]. The early detection of ischemia can be achieved by quantifying systolic and diastolic tissue velocities using TDI, which can also derive the strain and the strain rate [22]. The sensitivity of this tool makes it more effective in detecting early or minor myocardial abnormalities in ACS, potentially leading to faster diagnosis and intervention.

Recently, the EAS index, a parameter derived from TDI that comprehensively assesses diastolic and systolic function, calculated as [E’/(A’ *×* S’)], has been proposed for the prognostic stratification of patients with ischemic heart disease [19,23]. In a population of 415 patients with obstructive CAD, an elevated EAS index (i.e., ≥9.15) was significantly associated with a higher incidence of MACEs, including myocardial infarction, stroke, readmission for HF, coronary revascularization, and cardiovascular death within 6 months from first admission, particularly in those with an LVEF ≥ 50% [19,23].

## 5. TDI in Acute Heart Failure (AHF)

The European Society of Cardiology defines heart failure (HF) as a clinical syndrome characterized by typical symptoms and signs due to a structural and/or functional abnormality of the heart, resulting in increased intracardiac pressures and/or inadequate cardiac output at rest and/or during exercise [24]. Systolic or diastolic dysfunction, or both, can cause HF. Acute heart failure (AHF) is characterized by the rapid and progressive onset of symptoms and signs, often necessitating urgent medical evaluation and often leading to unplanned hospital admissions. The clinical presentation is variable, ranging from acute decompensated HF, acute pulmonary edema, and cardiogenic shock.

Echocardiography is recommended as the primary diagnostic tool for the assessment of cardiac function in patients with suspected acute and chronic HF [25]. It should provide the LVEF as a traditional measure of systolic function, along with other relevant parameters, including chambers size, regional wall motion abnormalities, diastolic, RV, and valvular function, pulmonary pressure, and signs of congestion [25].

Beyond the diagnosis, echocardiography findings are also useful in identifying some etiologies of AHF, as proposed by the guidelines through the acronym CHAMPIT, including ACS, hypertensive emergencies, arrhythmias, acute mechanical causes, acute pulmonary embolism, infection, and tamponade [25].

**Figure 2:**
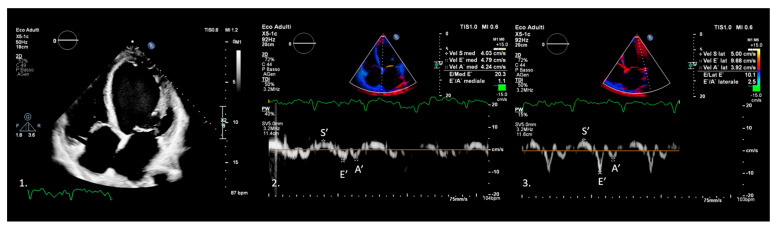
Tissue Doppler imaging (TDI) of a patient with dilated cardiomyopathy (1) showing a marked reduction in systolic and diastolic velocities at the inferior septum (2) and lateral wall (3) of the left ventricle, consistent with impaired systolic and diastolic function.

Even in this context, TDI provides valuable information (Figure 2), especially in estimating LV filling pressure. Classic echocardiography and tissue Doppler imaging (TDI) each contribute unique insights when evaluating acute heart failure, and their differences can be appreciated by considering how they approach the heart’s structure and function.

Classic echocardiography has traditionally focused on the heart’s overall anatomy and global function. Clinicians rely on it to assess chamber sizes, wall motion, and the ejection fraction, which are crucial for rapidly identifying significant abnormalities in an emergency scenario. This method in AHF offers a solid, visual representation of the heart’s structural integrity and gross performance. Yet because this technique largely relies on subjective interpretation—such as judging wall motion visually—it may not capture more subtle functional impairments, especially in instances where global measures like the ejection fraction appear normal [26]. TDI measures the velocity of the myocardial tissue during its contraction and relaxation phases. More sensitive detection of dysfunction, particularly diastolic abnormalities, can be achieved by focusing on the tissue velocities [27]. For example, TDI can quantify the early diastolic velocity (commonly referred to as E’), which when combined with traditional transmitral flow measurements (E), helps with calculating the E/E’ ratio, which is a useful estimate of left ventricular filling pressures. These measurements have been proven to be highly valuable in AHF, where the early detection of subtle dysfunction can be crucial for guiding therapeutic decisions [28]. While conventional echocardiography remains indispensable for assessing overall cardiac structure and for identifying obvious mechanical issues such as significant valvular disease or gross systolic dysfunction, TDI complements this evaluation by revealing finer details about myocardial performance. In the event of AHF, where rapid and precise management is crucial, TDI offers additional quantitative data that can have an immediate impact on the treatment decisions. By combining these techniques, a comprehensive diagnostic picture is created that combines structural assessments with dynamic, function-based insights, resulting in improved overall management of patients with AHF [29].

As already mentioned, a septal E’ velocity < 7 cm^2^ and an average E/E’ ratio > 14, along with an increased left atrial volume and tricuspid regurgitation velocity, are supportive of the presence of diastolic dysfunction and elevated LV filling pressure [30]. If the LVEF is preserved, these parameters can be beneficial in patients with dyspnea and signs of congestion [31,32].

The E/E’ ratio is accurate for the diagnosis of AHF, either in patients with reduced or preserved EF, maintaining a high diagnostic accuracy even in patients with inconclusive B-type natriuretic peptide (BNP) levels [33]. The E/E’ ratio and N-terminal pro-B-type natriuretic peptide (NT-proBNP) levels significantly correlate with the presence of B-lines on lung ultrasound [34,35], and were identified as independent predictors of cardiac death or hospitalization for worsening HF during a short follow-up in a cohort of patients admitted for acute or decompensated chronic HF (E/E’ ratio Hazard Ratio 1.047, 95% C.I. 1.006–1.090, *p* = 0.025; NT-proBNP HR 3.751, 95% C.I. 1.834 –7.767, *p* < 0.0001) [29]. According to a recent systematic review, mortality in critically ill patients was related to TDI indices of diastolic dysfunction [36,37].

Cardiogenic pulmonary edema is caused by an abrupt increase in cardiac filling pressures, which results in the rapid accumulation of fluid in the alveolar spaces. Diffuse alveolar filling without any elevation in the pulmonary capillary wedge pressure (PCWP) differentiates non-cardiogenic pulmonary edema [38]. Invasive PCWP measurements are typically used to make a differential diagnosis between these two conditions. Because it is invasive and has no significant impact on the prognosis, right heart catheterization is being performed less frequently in critically ill patients [39,40]. An elevated E/E’ ratio is widely accepted as a proxy for higher LV and pulmonary pressures [41]. The absence of Berlin criteria for ARDS and the presence of elevated LV filling pressures can be used to distinguish cardiogenic from non-cardiogenic pulmonary edema [42].

In the assessment of systolic HF, TDI offers a complementary method for semi-quantitative confirmation of systolic dysfunction by analyzing the S’ wave velocity [43]. Specifically, a cutoff value of 6.8 cm/s for the S’ velocity correlates with LV systolic dysfunction with an EF < 50% (sensitivity of 94.1% and specificity of 87%) [44].

Finally, TDI serves as a robust predictor of mortality. Specifically, S’ < 3 cm/s, E’ < 3 cm/s, A’ < 4 cm/s, and E/E’ > 20 were strongly associated with an increased risk of cardiac death within the following two years.

## 6. TDI in Hypertension

A hypertensive emergency (HE) is characterized by a sudden and severe increase in blood pressure that exceeds 180/120 mmHg. Assessing potential organ damage to the heart, brain, or kidneys is crucial when blood pressure exceeds these values [45]. Severe complications, such as acute intracerebral hemorrhage, ischemic stroke, or AHF, are connected to HEs.

The early identification of organ damage through a complete echocardiographic evaluation using TDI could have a significant impact on the clinical management and treatment strategies for these patients. Left ventricular hypertrophy, chamber dilatation, or wall motion abnormalities can be identified through classic echocardiography. TDI is capable of discovering a ratio of E/E’ >15, which is often found in individuals with hypertension and associated end-organ damage [46]. The identification of subclinical damage through the E/E’ ratio may speculatively lead to a reclassification of hypertension emergencies, suggesting a more aggressive treatment strategy in this setting.

## 7. TDI in Septic Cardiomyopathy

The increasing use of bedside ultrasounds has led to the identification of multiple forms of septic-related heart disease in recent years [47]. Inflammatory activation and hemodynamic stress are two mechanisms that contribute to the pathogenesis of sepsis-related heart disease [48,49].

Right ventricle systolic dysfunction and left ventricle diastolic dysfunction can affect the prognosis of septic patients, alongside left ventricle systolic dysfunction [47,48,50]. Sanfilippo et al. performed an analysis of 636 patients with sepsis and septic shock and found that almost 50% had some degree of diastolic dysfunction, as confirmed through TDI [51]. Diastolic dysfunction resulted in almost twice the risk of mortality (RR 1.82; 95% CI 1.12–2.97). In contrast, this outcome was not influenced by systolic dysfunction. Based on this observation, it can be concluded that a comprehensive echocardiographic study which includes TDI is effective in identifying patients who are at increased risk of adverse events in whom closer monitoring and more targeted therapy may be advantageous [52]. In fact, classic echo typically evaluates global parameters, such as the ejection fraction and wall motion, without being able to capture the subtle, regional dysfunction that sepsis may induce. In contrast, TDI directly quantifies the velocities of the myocardial tissue, making it more sensitive to early impairments in both systolic and diastolic function. For instance, even if the ejection fraction appears normal in septic patients, TDI can reveal reduced systolic velocities (S′) and impaired diastolic relaxation (via a decreased E′ velocity) that indicate intrinsic myocardial depression [53]. The E/E′ ratio, which is derived by combining TDI and mitral inflow data, further helps estimate left ventricular filling pressures, which is pivotal in managing these critically ill patients. In the presence of an elevated filling pressure, it may be advantageous to avoid fluid overload and administer vasoactive agents early, as an example [54].

## 8. TDI in Atrial Fibrillation

Atrial fibrillation (AF) is a common heart rhythm disorder, and is linked to increased rates of hospitalization, complications and mortality, often due to coexisting medical conditions [55,56]. HF is one of the most common non-fatal outcomes in patients with AF, affecting approximately half of these individuals over time [57]. In fact, patients with AF have a four- to five- fold increase in the relative risk of HF compared to those without AF [58,59]. Ischemic stroke, ischemic heart disease, and other thromboembolic events are all common adverse outcomes [56,59,60].

In AF, one of the key challenges is assessing the extent of atrial remodeling and electrical conduction abnormalities, which are central to both the initiation and the maintenance of the arrhythmia. TDI can quantify atrial electromechanical delays by measuring parameters such as the PA-TDI duration, defined as the interval between the onset of the P-wave in an ECG and the peak in the atrial contraction velocity at the lateral mitral annulus. This measurement reflects the total atrial conduction time and offers a surrogate for the degree of atrial fibrosis and remodeling. An increased PA-TDI duration has been associated with a higher risk of developing AF and with a reduced likelihood of successful rhythm control interventions [61].

The second focus is on evaluating diastolic dysfunction, which is a common occurrence in patients with both AF and HF. Despite some potential pitfalls, such as variability in the R-R interval, echocardiography demonstrates good overall accuracy in detecting elevated LV filling pressures, especially in patients with AF [62]. In this context, a septal E/E’ ratio > 11 is one of the supportive parameters for diagnosing elevated LV filling pressures [63]. As emphasized by the guidelines, the lateral e′ velocity is more affected by R-R interval variability, with the use of septal e′ alone recommended for this reason [63].

In addition, the E/E’ ratio is a valuable parameter for stratifying the risk of AF recurrence and assessing the associated risk of morbidity and mortality [64]. Furthermore, by providing sensitive quantitative data that may not be apparent in conventional imaging, TDI helps clinicians stratify risk and monitor the progression of atrial remodeling better and potentially guides therapeutic strategies, such as the decisions regarding rhythm control versus rate control or the need for more intensive monitoring post-ablation. The underlying atrial substrate that predisposes patients to AF can be refined further using TDI, which complements the standard echocardiographic measures [65] An independent association between higher LV filling pressures and an increased risk of death was found in a large study of patients with AF who underwent baseline transthoracic echocardiography [66]. An E/E’ ratio of 13 or higher significantly improves the prediction of death beyond other clinical factors [66]. Furthermore, the E/E’ ratio can predict left atrium appendage thrombi, neurological events, and cardiovascular events with high accuracy beyond the CHA2DS2-VASC score [67,68]. The E/E’ ratio also predicts the risk of AF recurrence after electrical cardioversion and catheter ablation [69,70]. In a study enrolling 127 patients with persistent AF, a septal E/E ratio of ≥11 was a predictor of AF recurrence, along with a duration of AF > 90 days before cardioversion [59,60]. Similarly, an average E/E’ ratio > 13 was associated with an increased recurrence rate at 12 months in a cohort of 198 patients with a normal LVEF who underwent catheter ablation [69,70].

Measurements of the S’ and E’ velocities have been shown to have a prognostic relevance in patients with HF with a preserved EF and AF, along with the E/E’ ratio. In a study by Shin et al. S’ velocity < 5 cm/s and E’ < 7 cm/s were independently associated with the risk of cardiovascular death, recurrent HF, and ischemic stroke, particularly when both present (HR 12.2, 95% CI 1.62–92.5; *p* = 0.015) [71].

## 9. TDI in Acute Pulmonary Embolism

Venous thromboembolism (VTE), which manifests clinically as deep vein thrombosis or pulmonary embolism (PE), is the third most common acute cardiovascular disorder worldwide, after myocardial infarction and stroke [67]. PE has an annual incidence rate of 39 to 115 cases per 100,000 individuals, with a rise in incidence rates and a decline in mortality [72]. Approximately half of all PE cases are diagnosed in emergency settings, where timely identification and treatment have been shown to significantly improve outcomes and increase survival rates [73].

**Figure 3:**
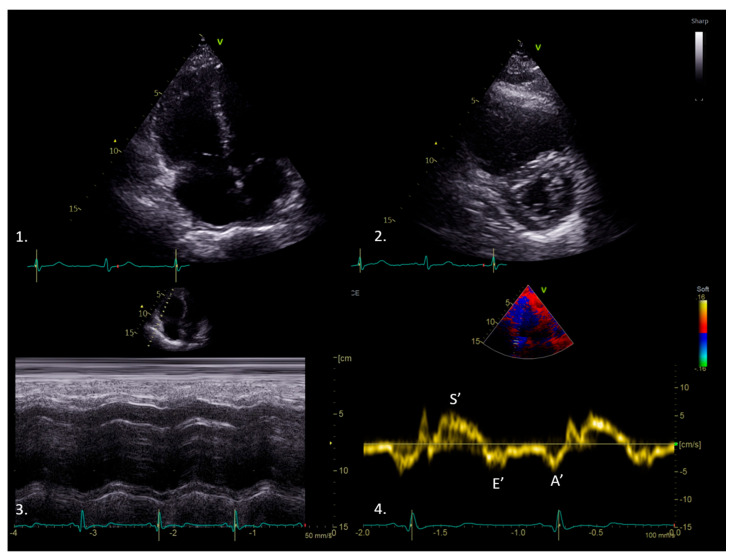
Transthoracic echocardiography of a patient with massive pulmonary embolism showing right ventricular dilatation (1); signs of pressure overload (2); reduced longitudinal function, as demonstrated by a reduced tricuspid annular plane systolic excursion (3); and reduced systolic velocity (S’) of the right ventricular lateral wall in tissue Doppler imaging (4).

In the context of PE, an acute increase in pulmonary vascular resistance can strain the right ventricle (RV), making its functional assessment crucial. Echocardiography shows several typical findings in PE (Figure 3), including RV dilatation, paradoxical motion of the interventricular septum, the “60/60 sign” (i.e., pulmonary flow acceleration time < 60 msec with a tricuspid regurgitation pressure gradient < 60 mmHg), and the presence of right heart thrombi [67,74]. Furthermore, acute PE may cause global RV hypokinesia, with preserved apical contraction (i.e., McConnell’s sign). If TAPSE values are lower than 16 mm, particularly with elevated pulmonary pressures, this can indicate right systolic dysfunction linked to PE [67,74]. However, traditional echocardiography is typically used to detect gross changes in the RV—such as dilation, interventricular septal flattening, and a decreased RV fractional area—thereby providing an overall assessment of RV strain and potential hemodynamic compromise. TDI can measure the RV’s peak systolic velocity (S′) in an acute PE setting; if S′ decreases, impaired systolic function is suspected, even if the conventional measurements are borderline [75] The S’ velocity (Figure 3) in TDI can be used as an indicator of RV systolic function and has a moderate correlation with the MRI-derived RVEF [76].

A value less than 9.5 cm/s is regarded as abnormal and is associated with RV systolic dysfunction [75,77]. The sensitivity of TAPSE and TDI as standalone indicators is limited. However, a reduced S’ velocity in PE is associated with a worse prognosis, even if it is not an independent predictor of mortality [72].

RV diastolic dysfunction can be detected using TDI in patients with a recent history of PE. In a study by Dentali et al., RV diastolic dysfunction, defined by an E’/A’ ratio < 1, was observed in more than half of the patients with a recent PE [76].

Another TDI-derived parameter is the Myocardial Performance Index (MPI), which is calculated as the time interval from the onset of isovolumic contraction to the end of isovolumic relaxation, divided by the ventricular ejection time. The MPI is significantly higher in patients with PE, primarily due to the prolongation of IVRT. MPI values greater than 0.55 have a sensitivity of 85% and a specificity of 78% in identifying PE [78,79]. Another useful parameter is called the M index, which consists of the peak early diastolic mitral inflow velocity divided by the RV MPI. The diagnosis of PE is highly sensitive and specific when these values are below 112 [73]. These values are useful also during follow-up, as they tend to normalize one month following effective treatment [74]. The MPI is also valuable for differentiating patients with pulmonary arterial hypertension (PAH) from those with acute PE, with these values being lower in the latter group [73,74].

## 10. TDI in Constrictive Pericarditis

Constrictive pericarditis (CP) is a condition in which a thickened and stiff pericardium restricts the normal filling of the heart chambers, ultimately leading to heart failure due to increased filling pressures and decreased cardiac output [80]. Several causes have been described, including viral and bacterial infections (e.g., tuberculosis), previous chest radiation therapy, and cardiac surgery [81]. Elevated ventricular filling pressures and interdependence of the ventricular system must be demonstrated for diagnosis [8].

Echocardiography is the first-line imaging technique in patients with suspected CP. While the classic echocardiographic techniques rely on features such as septal bounce and respiratory variations in ventricular filling to suggest constriction, TDI can offer a more quantitative assessment of myocardial motion. TDI is an essential tool for echocardiographic evaluation, revealing some distinctive signs [82]. Early diastolic velocity (E’) in TDI is a crucial measure that can be measured at both the basal septum and the lateral wall of the LV. In CP, the septal E’ velocity is generally maintained or even increased, while the lateral E’ velocity is usually decreased [83]. This pattern, known as annulus reversus, occurs because the constricting pericardium delays the movement of the lateral wall, while the septal E’ velocity increases due to the compensatory exaggerated longitudinal motion of the interventricular septum [84]. Another distinctive sign is a normal E/E ratio despite the presence of elevated LV filling pressures, due to a normal or an increased septal e’ velocity, which is known as annulus paradoxus. In patients with CP, the E/E’ ratio and PCWP have been found to have an inverse correlation [85].

Another important factor in TDI analyses of CP is the assessment of respiratory variations in myocardial velocities. The respiratory cycle causes variations in intrathoracic pressure, which affect the dynamics of ventricular filling. These variations can be captured using TDI, with CP displaying exaggerated respiratory changes in E’ velocities, which are higher during expiration [86].

By combining transmitral and trans-tricuspid pulsed-wave Doppler studies, the diagnosis is made more refined because it reveals a restrictive filling pattern and significant respiratory variations in the early filling (E) flow velocity [81].

## 11. TDI in the Weaning from Invasive Mechanical Ventilation

While the criteria for initiating mechanical ventilation, whether invasive or non-invasive, are well-established, the criteria for weaning remain poorly defined. Early weaning can result in reintubation due to failure to wean. On the other hand, patients with respiratory failure who receive prolonged mechanical ventilation have an increased mortality rate [87,88,89].

The complex interactions between the heart and lungs that occur during ventilation can lead to weaning failure, and cardiac dysfunction is the most common cause [90]. The promise of TDI in this context is its sensitivity; detecting early impairments in contractile performance may help predict which patients are likely to succeed with breathing independently once extubated and which may still be at risk of weaning failure [91]. Failure to wean was significantly correlated with a lower E’ velocity and a higher E/E’ ratio, along with a higher E wave velocity [92]. Interestingly, the left ventricle ejection fraction, unlike these parameters, is not associated with success or weaning failure [91,92]. The easy repeatability of TDI studies makes these parameters particularly useful for monitoring individual patients over time, aiding in the determination of the optimal timing for successful extubation. Its widespread adoption is currently hindered by technical challenges, such as maintaining an optimal angle of insonation and standardizing the measurement techniques.

TDI provides an intriguing and quantitative method for evaluating diaphragmatic performance while weaning from mechanical ventilation [93]. In this setting, the technique involves using ultrasound to measure the velocity of diaphragmatic motion—essentially capturing how quickly the diaphragm contracts [94]. Quantitative data can be particularly useful in identifying subtle diaphragmatic dysfunctions that may not be evident when using the conventional ultrasound methods, like simply assessing diaphragmatic excursion or the thickening fraction [95].

## 12. Discussion

TDI is a valuable tool in clinical practice because it has multiple advantages across a range of acute clinical scenarios, as highlighted in Table 2. TDI’s strengths are particularly evident in time-sensitive environments such as emergency medicine, where clinicians can obtain critical functional data on demand through its rapid execution and accessibility. At its core, TDI leverages the Doppler principle to measure the velocity of the myocardial and diaphragmatic tissues [7,8], rather than measuring blood flow. This offers a high temporal resolution that is essential for detecting subtle changes in cardiac mechanics, such as minor impairments in contractility or early diastolic dysfunction. For example, while conventional echocardiography provides a qualitative—and sometimes subjective—assessment of global cardiac function (such as the chamber dimensions and overall wall motion), TDI delivers precise, numerical values. Parameters such as systolic tissue velocity (S′) and early diastolic velocity (E′) allow for an objective evaluation of systolic and diastolic function, respectively. More importantly, the calculated E/e′ ratio serves as an effective non-invasive index of left ventricular filling pressures [11,31], which are often altered in early disease states. TDI’s quantitative capability enables the detection of early functional abnormalities [9,10,20] before they become clinically apparent or cause overt dysfunction. The clinical impact of TDI is demonstrated through its use in acute myocardial infarction [18,20]. Local regions of the myocardium may have impaired contractility despite a preserved ejection fraction, and this can be detected through a reduction in S′ velocities. Early detection is crucial for risk stratification, as it can identify at-risk myocardial segments before permanent damage occurs, which allows for prompt and targeted intervention. Another setting in which TDI makes a significant difference is acute heart failure [24,25,28,29]. In many cases, patients with heart failure may have a normal global systolic function but experience substantial diastolic dysfunction. A reduction in E′ velocity, together with an elevated E/E′ ratio, provides clear evidence of impaired relaxation and increased filling pressures. These findings not only help differentiate the severity and type of heart failure but also inform the decisions regarding therapeutic adjustments—whether tailoring diuretic regimens or optimizing the afterload reduction to manage the hemodynamic state better. Beyond left ventricular evaluations, TDI’s utility extends to assessing other critical functions. Measurements of the tricuspid annular systolic velocity (S′) are a sensitive indicator of right ventricular dysfunction in pulmonary embolism [67,74,75], for instance. TDI can be utilized to measure the tissue velocity and contractile performance of the diaphragm during mechanical ventilation weaning. By identifying subtle diaphragmatic dysfunctions that may not be evident when using the standard ultrasound techniques [93,94], this application may aid in predicting weaning success or failure. TDI has an additional advantage in its repeatability and reproducibility. Because TDI generates numerical, observer-independent data, clinicians can reliably monitor changes over time [9,10]. When managing critically ill patients, these serial assessments are invaluable because they allow for the continuous evaluation of therapeutic interventions and the early detection of evolving dysfunction. In summary, TDI has emerged as a powerful adjunct to conventional echocardiography by providing detailed, real-time quantitative insights into both myocardial and diaphragmatic performance. Whether it is utilized to detect early regional dysfunction in myocardial infarction, uncover diastolic abnormalities in acute heart failure [24,25,28,29], evaluate the right ventricular response in pulmonary embolism, or assess diaphragmatic function during weaning from ventilatory support, TDI enriches clinicians’ diagnostic and monitoring toolbox. In acute care settings, TDI will become a more important component of rapid, diagnosis-driven management, leading to more timely and effective patient care as the technology advances and standardization improves.

### 12.1. Limitations

TDI provides valuable quantitative insights into myocardial function, but it has several inherent limitations that must be considered in clinical practice.

The primary obstacle is its dependency on the angle. The accuracy of TDI measurements is dependent on the ultrasound beam being aligned with the direction of myocardial motion. Its diagnostic accuracy can be affected by deviations from this optimal alignment, leading to significant underestimations of velocities.

TDI’s sensitivity to noise and artifacts is also a challenge. Cardiac motion, adjacent tissue interference, or movement by the patient can affect the Doppler signals acquired using TDI. High-quality image acquisition and adequate filtering during post-processing are necessary, which can limit the reliability of the measurements in some clinical settings.

TDI parameters are also affected by the load dependency. The measured velocities are influenced not only by the intrinsic contractile properties of the myocardium but also by loading conditions such as the preload and afterload. In conditions where these loading factors are unstable, the interpretation of TDI-derived data can become complex.

Regional variability and tethering effects pose interpretative challenges as well. The motion recorded at a specific myocardial site can be affected by the movements of neighboring segments, which sometimes makes it difficult to isolate local dysfunction from more global effects.

Finally, this technique is operator-dependent and has not been fully standardized across different echocardiographic systems. The reproducibility of its findings may be reduced by interobserver variability caused by variations in the acquisition protocols, instrument settings, and analysis methods.

### 12.2. Future Directions

The future of TDI in acute clinical settings looks promising and diverse. The integration of TDI with artificial intelligence and automated analyses is a major focus of researchers and clinicians. Machine learning algorithms can be integrated into echocardiographic platforms to automatically detect and quantify subtle changes in myocardial tissue velocities [96,97]. Both the speed and accuracy of diagnosing acute conditions, such as myocardial ischemia and early signs of diastolic dysfunction, could be enhanced by this. Another promising direction involves combining TDI with advanced imaging modalities, such as integrating 3D echocardiography with TDI. By using this hybrid approach, clinicians could obtain volumetric assessments of myocardial function beyond the two-dimensional plane. A complete analysis of regional and global ventricular performance could be achieved through such integration, which is particularly necessary when emergency scenarios require rapid, precise evaluations [98,99]. Standardization and reproducibility improvements are also on the horizon. TDI’s angle dependency and variability between systems are currently causing challenges. Ongoing research is aimed at establishing more uniform protocols and threshold values that would facilitate its broader use in acute care and improve the interobserver consistency [100,101]. Enhanced standardization would validate TDI further as a reliable tool for rapid assessment of patients in the emergency department or an intensive care unit. Additionally, as portable and point-of-care ultrasound technologies evolve, the incorporation of TDI into these devices is expected to expand. This will enable more widespread, bedside applications in critical settings such as acute heart failure, sepsis, and pulmonary embolism and even in trials of weaning from mechanical ventilation [102]. The ability to deploy TDI quickly and efficiently at a patient’s bedside could streamline decision making and fine-tune management in real time.

## 13. Conclusions

Although it is considered an advanced echocardiographic technique, TDI could be of great benefit in the emergency setting, improving diagnoses, guiding therapeutic decisions, and providing valuable prognostic information.

## Figures and Tables

**Table 1:** Advantages and limitations of TDI in critically ill patients.

Advantages	Limitations
Easily and rapidly performed. Bed-sided examination. Reproducible. Quantitative. Diagnostic and prognostic information.	Acoustic window dependency. Angle dependency. Operator training and experience. It does not distinguish between active contraction and passive myocardial motion.

**Table 2:** A summary of the applications of TDI in major acute conditions and the level of evidence.

Clinical Condition	Clinical Impact	Performance	Cutoff Value	LOE
Acute coronary syndrome	Diagnosis of systolic and diastolic dysfunction	Early identification of acute ischemia (sensitivity 75–90%; specificity 80–90%)	Reduction of S’ > 30% from basal value E’ < 6 cm/s A’ > 10 cm/s	Level 4
	Prognosis	Reduction of S’ correlates with increased overall mortality (HR 1.5–2)	S’ < 5 cm/s	Level 4
Acute heart failure	Diagnostic of systolic or diastolic dysfunction.	S’ correlate with LVEF < 50% with sensibility of 94% and specificity of 87%.	- Systolic dysfunction S’ < 6.8 cm/s - Diastolic dysfunction E/E’ > 15	Level 4
	Diagnosis of acute heart failure.	- Preserved LV systolic function AUC = 0.875 - Reduced LV systolic function AUC = 0.903	- Preserved LV systolic function E/E’ > 10 - Reduced LV systolic function E/E’ > 15	Level 4
	Prognosis	High risk of cardiac death after 2 years	S’ < 3 cm/s E’ < 3 cm/s A’ < 4 cm/s E/E’ > 20	Level 4
Cardiogenic pulmonary edema	In patients with pulmonary edema, the E/E’ ratio is useful for the diagnosis of cardiogenic edema vs. ARDS	ND	E/E’ > 15	Level 6
Hypertensive crisis	In patients with hypertensive crisis, the E/E’ ratio is significantly higher in patients with hypertensive emergency compared to hypertensive urgency.	Urgency group vs. emergency group: 12.56 vs. 15.12 (*p* = 0.021)	>15	Level 4
Septic cardiomyopathy	Diastolic dysfunction is directly correlated with a higher mortality in sepsis	OR = 1.42 (95%CI: 1.14–1.76)	ND	Level 1
Acute pulmonary embolism	In patients presenting with PE S’ is a marker of RV systolic function. MPI and M index are useful for diagnosis.	- S’ shows moderate correlation with MRI-derived RVEF. - MPI have a sensitivity and specificity of 85% and 78% - M index have a sensitivity and specificity of 92%	S’ < 9.5 cm/s MPI > 0.55 M index < 112	Level 4
Atrial fibrillation	E/E’ ratio helps stratify the risk of AF recurrence, morbidity, and mortality	Recurrence: OR = 3.70, (95%95: 1.21–11.3); OR = 3.25 (95%CI: 1.19–8.86) Morbidity: OR = 1.64 (95%CI: 1.05–2.55); AUC = 0.83 (95%CI: 0.75–0.91) Mortality: HR = 1.32 (95% CI: 1.08–1.61)	Recurrence: E/E’ > 13/≥11 Morbidity: >15 Mortality: >13	Level 4 (Morbidity) Level 3 (Mortality)
Constrictive pericarditis	Diagnosis	Annulus reversus has a sensitivity of 70–90% and a specificity of 80–90% in diagnosing constrictive pericarditis.	Lateral E’ < Medial E’ (annulus reversus) Low E/E’ ratio despite high ventricular filling pressure (annulus paradoxus) E’ > 8 cm/s (differentiate from restrictive cardiomyopathy)	Level 4
Weaning from invasive mechanical ventilation	Weaning failure becomes more likely when the E/E’ ratio is high.	SMD = 1.70 (95%CI: 0.78–2.62)	ND	Level 1

Legend: AUC, area under curve; CI, confidence interval; HR, hazard ratio; LOE, level of evidence; LV, left ventricular; LVEF, left ventricular ejection fraction; ND, no date; OR, odd ratio; RCT, randomized controlled trial; SMD, standard mean difference. Levels of evidence (Melnyk and Fineout-Overholt): level 1, evidence from a systematic review or a meta-analysis of all relevant RCTs (randomized controlled trials); level 2, evidence from at least one well-designed RCT (e.g., large multi-site RCT); level 3, evidence from a single well-designed controlled trial without randomization (aka quasi-experimental studies) OR a systematic review of a complete body of evidence (an integrative review of higher and lower evidence) OR mixed-methods intervention studies; level 4, evidence from well-designed case–control or cohort studies; level 5, evidence from systematic reviews of descriptive and qualitative studies (meta-synthesis); level 6, evidence from a single descriptive or qualitative study, evidence-based projects, or evidence-base quality improvement and quality improvement projects; level 7, evidence from the opinion of authorities and/or reports of expert committees, reports from committees of experts, and narrative and literature reviews.
